# Previous experience of family violence and intimate partner violence in pregnancy

**DOI:** 10.11606/S1518-8787.2017051006700

**Published:** 2017-09-11

**Authors:** Ana Bernarda Ludermir, Thália Velho Barreto de Araújo, Sandra Alves Valongueiro, Maria Luísa Corrêa Muniz, Elisabete Pereira Silva

**Affiliations:** I Pós-Graduação em Saúde Coletiva. Centro de Ciências da Saúde. Universidade Federal de Pernambuco. Recife, PE, Brasil; II Departamento Materno Infantil. Centro de Ciências da Saúde. Universidade Federal de Pernambuco. Recife, PE, Brasil

**Keywords:** Pregnant Women, Violence Against Women, Domestic Violence, Intimate Partner Violence, Case-Control Studies

## Abstract

**OBJECTIVE:**

To estimate differential associations between the exposure to violence in the family of origin and victimization and perpetration of intimate partner violence in pregnancy.

**METHODS:**

A nested case-control study was carried out within a cohort study with 1,120 pregnant women aged 18–49 years old, who were registered in the Family Health Strategy of the city of Recife, State of Pernambuco, Brazil, between 2005 and 2006. The cases were the 233 women who reported intimate partner violence in pregnancy and the controls were the 499 women who did not report it. Partner violence in pregnancy and previous experiences of violence committed by parents or other family members were assessed with a standardized questionnaire. Multivariate logistic regression analyses were modeled to identify differential associations between the exposure to violence in the family of origin and victimization and perpetration of intimate partner violence in pregnancy.

**RESULTS:**

Having seen the mother suffer intimate partner violence was associated with physical violence in childhood (OR = 2.62; 95%CI 1.89–3.63) and in adolescence (OR = 1.47; 95%CI 1.01–2.13), sexual violence in childhood (OR = 3.28; 95%CI 1.68–6.38) and intimate partner violence during pregnancy (OR = 1.47; 95% CI 1.01 – 2.12). The intimate partner violence during pregnancy was frequent in women who reported more episodes of physical violence in childhood (OR = 2.08; 95%CI 1.43–3.02) and adolescence (OR = 1.63; 95%CI 1.07–2.47), who suffered sexual violence in childhood (OR = 3.92; 95%CI 1.86–8.27), and who perpetrated violence against the partner (OR = 8.67; 95%CI 4.57–16.45).

**CONCLUSIONS:**

Experiences of violence committed by parents or other family members emerge as strong risk factors for intimate partner violence in pregnancy. Identifying and understanding protective and risk factors for the emergence of intimate partner violence in pregnancy and its maintenance may help policymakers and health service managers to develop intervention strategies.

## INTRODUCTION

Violence inside the family context has been presented as a phenomenon with high frequencies^2^ ^,^ ^28^ and passed down through the generations^33^. Exposure to intimate partner violence (IPV) in childhood or adolescence can be associated with IPV perpetration or victimization in adulthood^28^. The complex, multidimensional nature of family violence has been identified in international^12^ and Brazilian studies^4^ ^,^ ^35^, which have demonstrated a co-occurrence of different forms of violence.

Intimate partner violence during pregnancy (IPVP) is a social and public health problem, both in its magnitude and consequences, with short^21^ and long-term outcomes for women and children^23^.

In a review of studies conducted in developed and developing countries, Taillieu and Brownridge^33^ have identified variations in the prevalence of IPVP. Psychological violence ranged between 1.5% and 43.2%, physical violence between 0.9% and 30%, and sexual violence between 1% and 3.9%. In a systematic review of African studies^32^, the prevalence of IPVP ranged from 2% to 57%. Han and Stewart^19^ have found prevalence rates of IPVP in Latin America and the Caribbean, which ranged from 3% to 34.5% for sexual violence, 2.5% to 38.7% for physical violence, and 13% to 44% for psychological violence. In Brazil, physical or sexual violence has been found in 6.5% of pregnant women^5^, severe physical violence in 18.9%^25^ of them, and psychological violence ranged from 19.1%^5^ to 41.6%^29^.

Several theoretical perspectives attempt to explain intimate partner violence (IPV) in women’s lives^8^. However, the specificities of IPVP still remain poorly understood. Taillieu and Brownridge^33^ mention some explanations: stress related to pregnancy, social learning theory, and evolutionary psychology.

Some factors related to pregnancy may increase the stress of the couple and, therefore, increase the risk of violence, such as: primiparity and unwanted pregnancy^5^, economic difficulties^20^, and the change in the social role of women and men when becoming parents^22^.

Theorists of social learning postulate that behavior is shaped by behavioral models that the child observes within their family of origin^6^. The theory suggests that children exposed to IPV learn that aggression is either an appropriate strategy to manage stress and resolve conflicts or a way of obtaining control, both in intimate and social relationships^16^. Moreover, children with violent parents may not have the opportunity to learn socially positive methods of effective communication and conflict resolution, such as: negotiation, verbal reasoning, self-control tactics, and active listening^9^.

Children who grow up in a family that faces stress and frustration with anger and aggressiveness present greater risk of showing the same behavior, to which they were directly or indirectly exposed, when becoming adults^24^. Similarly, children in families in which there are both IPV and violence against them are not only exposed to IPV, but also to additional opportunities for learning aggressive behavior patterns^16^.

Some studies have shown that boys exposed to IPV are more likely to become perpetrators of IPV as adults, while girls are more likely to become victims as adults, in relation to children who were not exposed to IPV^34^. In the study of Black et al.^9^, interparental psychological violence was witnessed by most of the respondents (58.3%), who also experienced psychological violence in their own intimate relationship (69.5%). Similarly, physical violence was also witnessed among parents (17.5%) and experienced in their own intimate relationships (27.0%). Cannon et al.^11^ have found that 49% of children who had experienced their mother being abused by an intimate partner were the daughters of women who were also exposed to IPV in childhood.

Suffering repeated physical and psychological violence by relatives throughout life is a factor associated with IPVP. In a Brazilian study, Audi et al.^4^ have identified that 55.8% of women reported experience of violence during childhood. Among this number, 31.3% witnessed physical violence in the family, 17.8% were victims of physical violence, and 6.7% had experienced some form of sexual abuse. These exposures were associated with IPVP.

The emphasis of the social learning theory, which has been used to explain the intergenerational transmission of violent behavior, is on learning by observation, imitation, and modeling. Children become part of the intergenerational cycle of violence because they learn and incorporate the lessons of violence and, without intervention, grow and repeat this behavior^3^.

Evolutionary Psychology suggests that persons unconsciously struggle not only for personal survival but also for the perpetuation of their genetic legacy, which may explain the jealousy, possessiveness, and insecurity of male partners during pregnancy and IPVP^20^. These data are relevant to the understanding of violence against pregnant women^33^.

The objective of this study was to identify differential associations between the exposure to violence in the family of origin and victimization and perpetration of IPVP.

## METHODS

A nested case-control study was carried out within a cohort study designed to investigate risk factors for postnatal depression and adverse maternal and personal outcomes in Health District II (one of the six health areas) in Recife, Northeastern Brazil, between 2005 and 2006. The population of the health district was 217,293 inhabitants, which represented almost 15.0% of the population of Recife, and it has a high proportion of low-income families.

The study population consisted of all (1,133) pregnant women aged 18–49 years old in the third trimester of pregnancy registered in the Family Health Strategy (Health Family Program – HFP – and Community Health Worker Program). The coverage of the Family Health Program (FHP) was approximately 78.0% of the population. Baseline data for the cohort in our study have been reported elsewhere^21^.

Pregnant women were identified from antenatal care records from 42 primary care teams as well as from the records of community health workers in order to include those not receiving antenatal care at the Health Family Program units. Data were collected by trained female interviewers and most often performed at a healthcare unit. Some interviews were conducted in the interviewee’s home at the woman’s request.

The study achieved a high response rate (98.8%) and 1,120 of the 1,133 pregnant women were eligible for inclusion in the study, of whom 732 had complete data for all variables and were included in the analysis of the study.

The cases were the 233 (31.8%, 95%CI 28.5–35.3) women who reported some form of IPV during pregnancy and the controls were the 499 (68.2%, 95%CI 64.7–71.5) pregnant women who did not report IPVP.

The questions relating to violence were developed by the international team of the WHO multi-country study on women’s health and domestic violence^17^. As in all other countries, the Brazilian/Portuguese questionnaire was independently back-translated and discussed during interviewer training and piloting.

Intimate partners were defined as being the partner or ex-partner with whom the woman lived or used to live, regardless of formal union, including current partners with whom they maintained sexual relations. Therefore, women could report partner violence even if they were not with a partner at the time of the antenatal interview. To identify IPVP, the questions characterized physical violence as physical aggression or use of objects or weapons to produce injuries; psychological violence as threatening behavior, humiliation, and insults; and sexual violence as sexual intercourse imposed using physical force or threats and imposition of acts that were considered humiliating. The IPVP was considered positive if the woman answered “yes” to at least one of the questions that comprise each type of violence^21^.

The analysis of early experiences of violence was conducted with a theoretical-conceptual model (Figure) which describes the possible associations between the woman’s and the partner’s experiences of violence during childhood, adolescence, and adulthood, whether by witnessing their mothers suffer violence or by being victims or perpetrators of violence.

**Figure:**
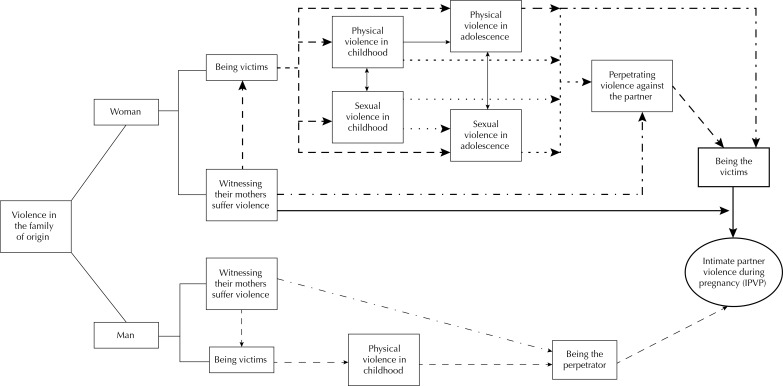
Theoretical and conceptual model of the possible stages from childhood violence to experiences of violence during pregnancy.

We investigated the variables described in the literature as being associated with IPVP: a) socioeconomic and demographic (age: 18–19 years *versus* ≥ 20 years, race: white *versus* non-white, living without a partner: yes *versus* no, years of schooling: 0–4 *versus* ≥ 5, house ownership: owned *versus* rented), own income: yes *versus* no, employment status: employed *versus* not employed); b) behavioral characteristics (aggressive behavior by the partner outside the home: yes *versus* no), and c) relationship profile of the couple (communication with the partner: good *versus* poor, controlling behavior of partner: not controlling *versus* moderate and very controlling, fights between the couple: yes *versus* no, women’s infidelity: yes *versus* no, and partner’s infidelity: yes *versus* no). Communication with the current or most recent partner and controlling behavior of partner were described elsewhere^21^.

Analysis was performed with Stata for Windows, version 10.1. Logistic regression analyses were modeled to identify differential associations between the exposure to violence in the family of origin and victimization and perpetration of IPVP. Variables that had p < 0.20 in the univariate analysis were included in the multivariate analysis. Odds ratios (OR) and 95% confidence intervals were calculated between IPVP and other exposures to violence.

The study received approval from the Ethics Committee of the Universidade Federal de Pernambuco (Protocol 303/2004). Confidentiality and privacy of the interviewees were guaranteed. All women gave written informed consent before taking part in the study. They all received information, specifically produced for this purpose, on social, health, legal, and police services available in the area under study regardless of the presence of partner violence. Services were contacted to assist those women interviewed who were in life-threatening situations.

## RESULTS

Table 1 presents the socioeconomic, demographic, and behavioral characteristics and the relationship profile of cases and controls. The IPVP were more likely in women without a partner, with lower education, in those who had a controlling partner and with aggressive behaviors outside the home, without communication with the partner, with fights with their partner, and in the presence of women’s and partner’s infidelity.

**Table 1:** Association of the socioeconomic, demographic, and behavioral characteristics and the relationship profile with intimate partner violence in pregnancy. Municipality of Recife, Northeastern Brazil, 2005–2006.

Variable	Cases		Controls		Total		Odds ratio	95%CI	p
	
	(n = 233)		(n = 499)						

	n	%	n	%	n	%			
Socioeconomic and demographic characteristics									

Women’s age									
18–19 years	34	14.6	72	14.4	106	14.5	1.00	0.65–1.57	0.95
≥ 20 years	199	85.4	427	85.6	626	85.5	1.00		
Partner’s age									
18–19 years	28	12.0	22	4.4	50	6.8	2.96	1.65–5.29	< 0.0001
≥ 20 years	205	88.0	477	96.0	682	93.2	1.00		
Women’s race									
Non-white	194	83.3	386	77.4	580	79.2	1.46	0.97–2.17	0.067
White	39	16.7	113	22.6	152	20.8	1.00		
Partner’s race									
Non-white	162	69.5	338	67.7	500	68.3	0.92	0.66–1.29	0.627
White	71	30.5	161	32.3	232	31.7	1.00		
Living without a partner									
Yes	36	15.5	35	7.0	71	9.7	2.42	1.47–3.97	< 0.0001
No	197	84.5	464	93.0	661	90.3	1.00		
Years of schooling									
0–4	66	28.3	82	16.4	148	20.2	2.00	1.39–2.91	< 0.0001
≥ 5	167	71.7	417	83.6	584	79.8	1.00		
House ownership									
Rented	83	35.6	161	32.3	244	35.4	1.16	0.83–1.61	0.37
Owned	150	64.4	338	67.7	488	64.6	1.00		
Own income									
Yes	127	54.5	277	55.5	404	52.2	0.96	0.70–1.31	0.79
No	106	45.5	222	44.5	328	44.8	1.00		
Women’s employment status									
Not employed	183	78.5	369	74.0	552	75.4	1.29	0.88–1.86	0.18
Employed	50	21.5	130	26.0	180	24.6	1.00		
Partner’s employment status									
Not employed	59	25.3	107	21.5	166	22.7	0.80	0.56–1.16	0.25
Employed	174	74.7	391	78.5	565	77.3	1.00		

Behavioral characteristics									

Aggressive behavior by the partner outside the home									
Yes	71	30.5	52	10.4	123	16.8	3.77	2.52–5.62	< 0.0001
No	162	69.5	447	89.6	609	83.2	1.00		

Relationship profile									

Communication with partner									
Poor	32	13.7	24	4.8	56	7.7	3.15	1.81–5.48	< 0.0001
Good	201	86.3	475	95.2	676	92.3	1.00		
Controlling behavior of partner									
Moderate and very controlling	208	89.3	305	61.1	513	70.1	5.29	3.36–8.31	< 0.0001
Not controlling	25	10.7	194	38.9	219	29.9	1.00		
Fights between the couple									
Yes	146	62.7	139	27.9	285	38.9	4.35	3.12–6.04	< 0.0001
No	87	37.3	360	72.1	447	61.1	1.00		
Women’s infidelity									
Yes	14	6.0	10	2.0	24	3.3	3.12	1.36–7.14	0.007
No	219	93.7	489	98.0	708	96.7	1.00		
Partner’s infidelity									
Yes	117	50.2	131	26.3	248	33.9	2.83	2.04–3.92	< 0.0001
No	116	49.8	368	73.7	484	66.1	1.00		

Table 2 shows the association of women’s experiences of violence in the family of origin and the perpetration of IPV, as well as victimization during pregnancy. The analysis of the first, second, fourth, and sixth stage reveals the situations of victimization of the women. In childhood, having seen the mother suffer IPV was associated with physical violence in childhood (OR = 2.62, 95%CI 1.89–3.63) and in adolescence (OR = 1.47, 95%CI 1.01–2.13), sexual violence in childhood (OR = 3.28, 95%CI 1.68–6.38), and IPVP (OR = 1.47, 95%CI 1.01–2.12). The IPVP was frequent in women who reported more episodes of physical violence in childhood and adolescence and sexual violence in childhood. The fourth stage shows that physical violence in childhood increased the chance of physical violence in adolescence (OR = 1.89, 95%CI 1.31–2.74) and sexual violence in childhood (OR = 2.48, 95%CI 1.28–4.79). Similarly, the chance of sexual violence in adolescence is increased by the experience of physical violence in adolescence (OR = 3.31, 95%CI 1.76–6.21) and sexual violence in childhood (OR = 2.84, 95%CI 1.07–7.53).The third, fifth, and seventh stage show the situations of victimization of women and the perpetration of violence against the partner. Physical violence in childhood (OR = 1.85, 95%CI 1.13–3.05) and adolescence (OR = 2.59, 95%CI 1.54–4:35) presented statistically significant association with the perpetration of violence against the partner, which, in turn, increased the chance of the women suffering IPVP (OR = 8.67, 95%CI 4.57–16.45).

**Table 2:** Association of women’s experiences of violence in the family of origin, the perpetration of intimate partner violence, and victimization of intimate partner violence in pregnancy. Municipality of Recife, Northeastern Brazil, 2005–2006.

Variable	Cases		Controls		Unadjusted OR	95%CI	p	Adjusted OR*	95%CI	p
	
	n	%	n	%						
1st stage										


Witnessing the mother suffer IPV and physical violence in childhood										
Yes	127	50.0	125	26.2	2.82	2.05–3.88	< 0.0001	2.62	1.89–3.63	< 0.0001
No	127	50.0	353	73.8	1.00			1.00		
Witnessing the mother suffer IPV and physical violence in adolescence										
Yes	69	43.4	183	31.9	1.63	1.14–2.34	0.007	1.47	1.01–2.13	0.041
No	90	56.6	390	68.1	1.00			1.00		
Witnessing the mother suffer IPV and sexual violence in childhood										
Yes	25	61.0	227	32.9	3.19	1.67–6.10	< 0.0001	3.28	1.68–6.38	< 0.0001
No	16	39.0	464	67.1	1.00			1.00		
Witnessing the mother suffer IPV and sexual violence in adolescence										
Yes	20	44.4	232	33.8	1.56	0.85–2.88	0.147	1.33	0.71–2.48	0.370
No	25	55.6	455	66.2	1.00			1.00		

2nd stage										

Witnessing the mother suffer IPV and IPVP										
Yes	100	42.9	152	30.5	1.72	1.24–2.40	0.001	1.47	1.01–2.12	0.042
No	133	57.1	347	69.5	1.00			1.00		

3rd stage										

Witnessing the mother suffer IPV and perpetrating violence against the partner without being assaulted first										
Yes	29	35.8	223	34.2	1.07	0.66–1.73	0.782	0.81	0.48–1.35	0.420
No	52	64.2	428	65.8	1.00			1.00		

4th stage										

Physical violence in childhood and physical violence in adolescence										
Yes	77	48.4	177	30.9	2.10	1.46–3.00	< 0.0001	1.89	1.31–2.74	0.001
No	82	51.6	396	69.1	1.00			1.00		
Physical violence in childhood and sexual violence in childhood										
Yes	23	56.1	231	33.4	2.54	1.35–4.80	0.004	2.48	1.28–4.79	0.007
No	18	43.9	460	66.6	1.00			1.00		
Physical violence in adolescence and sexual violence in adolescence										
Yes	22	48.9	137	19.9	3.84	2.07–7.09	< 0.0001	3.31	1.76–6.21	< 0.0001
No	23	51.1	550	80.1	1.00			1.00		
Sexual violence in childhood and sexual violence in adolescence										
Yes	6	13.3	35	5.1	2.86	1.13–7.22	0.026	2.84	1.07–7.53	0.036
No	39	86.7	652	94.9	1.00			1.00		

5th stage										

Physical violence in childhood and perpetrating violence against the partner without being assaulted first										
Yes	42	16.5	39	8.2	2.23	1.40–3.55	0.001	1.85	1.13–3.05	0.015
No	212	83.5	439	91.8	1.00			1.00		
Physical violence in adolescence and perpetrating violence against the partner without being assaulted first										
Yes	35	43.2	124	19.0	3.23	1.99–5.23	< 0.0001	2.59	1.54–4.35	< 0.0001
No	46	56.8	527	81.0	1.00			1.00		
Sexual violence in childhood and perpetrating violence against the partner without being assaulted first										
Yes	8	19.5	73	10.6	2.05	0.91–4.61	0.082	2.09	0.86–5.05	0.103
No	33	80.5	618	89.4	1.00			1.00		
Sexual violence in adolescence and perpetrating violence against the partner without being assaulted first										
Yes	8	9.9	37	5.7	1.82	0.81–4.05	0.144	1.37	0.58–3.23	0.471
No	73	90.1	614	94.3	1.00			1.00		

6th stage										

Physical violence in childhood and IPVP										
Yes	113	48.5	141	28.3	2.40	1.73–3.30	< 0.0001	2.08	1.43–3.02	< 0.0001
No	120	51.5	358	71.7	1.00			1.00		
Physical violence in adolescence and IPVP										
Yes	70	30.0	89	17.8	2.00	1.37–2.84	< 0.0001	1.63	1.07–2.47	0.023
No	163	70.0	410	82.2	1.00			1.00		
Sexual violence in childhood and IPVP										
Yes	25	10.7	16	3.2	3.63	1.89–6.93	< 0.0001	3.92	1.86–8.27	< 0.0001
No	208	89.3	483	96.8	1.00			1.00		
Sexual violence in adolescence and IPVP										
Yes	22	9.4	23	4.6	2.20	1.17–3.95	0.013	1.72	0.87–3.41	0.119
No	211	90.6	476	95.4	1.00			1.00		

7th stage										

Perpetrating violence against the partner without being assaulted first and IPVP										
Yes	66	28.3	15	3.0	12.75	7.08–22.94	< 0.0001	8.67	4.57–16.45	< 0.0001
No	167	71.7	484	97.0	1.00			1.00		

IPV: Intimate partner violence; IPVP: Intimate partner violence in pregnancy

* Adjusted for race, living without the partner, years of schooling, employment status, aggressive behavior by the partner outside the home, communication with partner, controlling behavior of partner, fights between the couple, women’s infidelity, and partner’s infidelity.

Regarding the partner, Table 3 shows that having witnessed the mother suffer IPV in his childhood was associated with physical violence in childhood (OR=2.83, 95%CI 2.00–4.00) and perpetration of IPVP (OR=1.86, 95%CI 1.27–2.72). Having been a victim of physical violence in childhood increased the chance of perpetrating IPVP (OR=1.76, 95%CI 1.21–2.54).

**Table 3:** Association of partner’s experiences of violence in the family of origin and the perpetration of intimate partner violence in pregnancy. Municipality of Recife, Northeastern Brazil, 2005–2006.

Variable	Cases		Controls		Unadjusted OR	95%CI	p	Adjusted OR*	95%CI	p
	
	n	%	n	%						
1st stage										

Witnessing the mother suffer IPV and physical violence in childhood										
Yes	112	42.6	99	21.1	2.77	1.99–3.85	< 0.0001	2.83	2.00–4.00	< 0.0001
No	151	57.4	370	78.9	1.00			1.00		

2nd stage										

Witnessing the mother suffer IPV and perpetration of IPVP										
Yes	93	39.9	118	23.7	2.14	1.53–2.99	< 0.0001	1.86	1.27–2.72	0.002
No	140	60.1	381	76.3	1.00			1.00		

3^rd^ stage										

Physical violence in childhood and IPVP										
Yes	110	47.2	153	30.7	2.02	1.47–2.78	< 0.0001	1.76	1.21–2.54	0.003
No	123	52.8	346	69.3	1.00			1.00		

IPV: Intimate partner violence; IPVP: Intimate partner violence in pregnancy

* Adjusted for partner’s age, living without the partner, women’s years of schooling, women’s employment status, aggressive behavior by the partner outside the home, communication with partner, controlling behavior of partner, fights between the couple, women’s infidelity, and partner’s infidelity.

## DISCUSSION

The results indicate that experiences of violence in intimate relationships, including in times of emotional and physical vulnerability for women (such as pregnancy) were more frequent in women who reported violence in the family of origin, including witnessing the mother suffer IPV and being a victim of physical and/or sexual violence in childhood and physical violence in adolescence. A pattern of continuity has been identified, which has also increased the chance of perpetration of physical violence against the partner and IPVP.

Children who witness or experience violence are more likely to commit or be victims of violence when adults, when compared to children who were not exposed to violence^10^. Although it is clear that violence in the family of origin has an impact on perpetration and victimization, this relationship has not been fully explored regarding the specific risk of IPVP.

The IPVP is not an isolated incident in the life of a woman and may be part of a process of life. International studies have reported the association of IPV in life^2^ and IPVP^7^ ^,^ ^10^ ^,^ ^32^ with having witnessed the mother suffer violence and with having been a victim of physical or sexual violence in childhood. The IPV has also been found in a population-based survey conducted in Brazil with 15–49 years old women^13^. In Brazilian women who attended primary health care services, IPVP was associated with having witnessed the mother suffer violence and being a victim of physical violence in childhood^4^.

Witnessing the mother suffer IPV in childhood was associated with physical violence suffered by girls in childhood and adolescence. Studies have shown that when there is IPV in the family, children are more likely to be victims of physical violence because the men who assault their partners and the women who are involved in violent relationships abuse their children more often compared to the women who did not experience such violence^12^. One of the approaches to examine this potential association is the violent maternal educational practice^30^.

The association of IPVP with physical or sexual violence in childhood is consistent with a study carried out in the United States^26^. Pregnant women reporting some form of violence in childhood had a 2.5 times greater chance of experiencing IPVP. The pattern of continuity found between the experience of violence in childhood/adolescence and the association with IPVP can be explained by the risk of re-victimization of women who have been abused both physically and sexually in childhood^7^. Other studies have also concluded that women who witnessed IPV during childhood present a higher risk of experiencing IPV in adulthood^2^ ^,^ ^13^. Abeya et al.^1^ state that women who witness violence against their mothers are more likely to tolerate violence by their partners in a passive manner. Thus, it is possible that, in the future, these observers become silent victims of abuse. Furthermore, there is also evidence that witnessing or experiencing some form of abuse during childhood increases the risk of being a perpetrator of IPV in adulthood^11^.

Having been a victim of IPVP was approximately nine times more frequent among women who perpetrated physical violence against the partner. The experiences of violence in the family of origin showed lower percentages. However, they were collected from reports that require remembering events that occurred in childhood or adolescence, which are prone to recall bias. The perpetration of violence by women is more recent, thus less susceptible to this bias.

Perpetration of physical violence against the partner showed association with the experience of women as victims of physical violence in childhood or adolescence^14^. However, it has not been associated with sexual violence in childhood and/or adolescence, indicating that the consequences of exposure to physical violence in childhood and adolescence differ from those of sexual violence. The imitated behaviors also have gender-specific influence^18^ and they highlight the importance of the proposition of the social learning theory for modeling behaviors, based on direct and observed experiences in the family environment^14^.

In agreement with other studies^15^, having witnessed his mother be a victim of IPV, besides suffering physical violence in childhood, increases the chance of the partner perpetrating IPV, reinforcing previous research that suggests the intergenerational transmission of violence.

This study has several strengths. First, the large sample was recruited from family and community health workers programs with an excellent response rate, providing a representative community sample of poor persons in Recife. Second, we used an internationally recognized questionnaire that takes a non-judgmental approach to this sensitive subject. The questionnaire had its psychometric properties considered as adequate to estimate the occurrence of violence against women in Brazil^31^, and it has been used in several studies on IPVP^4^ ^,^ ^5^ ^,^ ^27^ ^,^ ^29^.

In addition, data were collected by interviewers experienced in addressing the issue of violence against women. Lastly, both cases and controls were obtained from the same cohort. Therefore, the controls represent the non-cases within the same reference population of the study. Thus, it may be considered that the results are representative of the population of pregnant women in Health District II.

Some limitations are also important to consider. First, the study setting and population could have biased the results of the study. The occurrence of partner violence is increased in women with little schooling and living in poverty, thus the high frequency of partner violence could be indicative of the characteristics of the community in our study.

To the best of our knowledge, despite the above mentioned limitations, this is the first study in Brazil to assess the association between IPVP and the history of woman’s and partner’s experiences of violence during childhood, adolescence, and adulthood.

Furthermore, violence could have been underreported because of the associated stigma and shame. Since this is a complex, delicate, and intimate topic, women may have had difficulties in recalling the traumatic situations as well as talking about them. This fact may have underestimated the findings of this study. Other factors, such as the relationship between the woman and the abuser, which involves fear or the desire to protect him, the place where the interview was conducted, the relationship between the interviewer and the interviewee, and uncertainty as to the confidentiality of their reporting, may also have underestimated the information concerning the partner’s violence^21^.

The high prevalence of IPVP reveals the magnitude of the problem in Recife. However, more research is needed to improve our understanding of the reality of this condition throughout Brazil, especially in population segments not represented in this sample. In addition to the magnitude of the problem, the associated risk factors and the impact that IPVP has on the lives of women and their children confirms that this is a public health problem.

Attention should be drawn to this multifactorial problem for which multi-sectoral interventions should be conducted in order to identify and prevent such abuse. Health and education systems play an important role in identifying domestic violence among children and adolescents and in protecting and empowering women who report IPV. Therefore, it is necessary to reinforce the importance of the discussion on gender equality in the curriculum and pedagogical planning of schools, even when considering that in 2014 the Brazilian Congress abolished the gender issue of the National Education Plan (PNE)^a^ in force until 2014. Health professionals also need to be trained and receive institutional support in order to track down and address the cases of violence against women. Interventions must also address childhood abuse and respond appropriately to children who have witnessed IPV^2^.

The results are a contribution to knowledge on IPVP, thus raising the awareness of health professionals regarding this subject, as well as the creation of prevention strategies to reduce the impacts on health. Therefore, it becomes imperative to intervene in this inter-generational cycle of abuse. We hope that current actions to reduce IPV and child abuse can decrease future occurrences of violence against women.

## Footnotes

a Brasil. Lei Nº 13.005, de 24 de junho de 2014 - Plano Nacional de Educação. Brasília (DF); 2014 [cited 2017 Jun 20]. Available from: http://fne.mec.gov.br/9-uncategorised/821-lei-n-13-005-de-24-de-junho-de-2014-plano-nacional-de-educacao
